# Lipid emulsion, but not propofol, induces skeletal muscle damage and lipid peroxidation

**DOI:** 10.1007/s00540-019-02676-8

**Published:** 2019-08-31

**Authors:** Tomohiro Chaki, Naoyuki Hirata, Yusuke Yoshikawa, Shunsuke Tachibana, Yasuyuki Tokinaga, Michiaki Yamakage

**Affiliations:** Department of Anesthesiology, Sapporo Medical University School of Medicine, 291, South 1, West 16, Chuo-ku, Sapporo, Hokkaido Japan

**Keywords:** Propofol, Lipid peroxidation, Intravenous fat emulsions, Antioxidants

## Abstract

**Purpose:**

Prolonged propofol infusion induces skeletal muscle damage. However, it is well known that the lipid emulsion that is the solvent of propofol causes various types of tissue damage via lipid peroxidation, and that propofol, conversely, has an anti-lipid peroxidative effect. The purpose of this study was to determine whether propofol or the lipid emulsion is the cause of muscle damage following prolonged administration.

**Methods:**

Rats were divided into four groups: NI group (no intervention), Cath group (venous catheter insertion only), Prop group (1% propofol (Maruishi) intravenous infusion at 10 mg**/**kg/h), and Lipid group (10% Lipofundin® intravenous infusion at 100 mg**/**kg/h) (*n* = 10, each group). 1% Propofol (Maruishi) or Lipofundin was infused at 1 mL/kg/h for 72 h. The solvent of 1% propofol (Maruishi) is a 10% lipid emulsion. Lipofundin consists of 50% long-chain triacylglycerols and 50% medium-chain triacylglycerols, similar to the propofol solvent. Plasma concentrations of creatine kinase and myoglobin, superoxide production level, and 4-hydroxynonenal and malondialdehyde expression in the gastrocnemius muscle were evaluated 72 h after the interventions.

**Results:**

Plasma concentrations of creatine kinase and myoglobin in the Lipid group were significantly higher than those in the other three groups. The superoxide production level, and 4-hydroxynonenal and malondialdehyde expression in the Lipid group were also significantly higher than in the other three groups.

**Conclusion:**

Lipofundin induces skeletal muscle damage via lipid peroxidation, and 1% propofol (Maruishi) conversely suppresses the muscle damage via antioxidant effects.

## Introduction

Propofol is a short-acting intravenous anesthetic that is frequently used as a sedative for patients receiving anesthesia and in intensive care units. Since it has high lipophilicity, propofol is dissolved in a lipid emulsion in clinical settings. However, it is well known that a high dose and prolonged duration of propofol infusion induce skeletal muscle damage [1, 2]. The mechanism of propofol-induced muscle damage is thought to be related to mitochondrial dysfunction, especially in the electron transport system on the inner mitochondrial membrane [3–5]. However, the details of the mechanism have not been clearly elucidated. Moreover, most studies investigated the effect of propofol itself, and the effect of the lipid emulsion that is the solvent of propofol has not been examined.

Similar to propofol, high doses of a lipid emulsion can also cause various types of tissue damage, including of the skeletal muscle [6]. Lipid emulsions contain large amounts of polyunsaturated fatty acids, such as soybean oil. In an in vivo experiment, intravenous administration of polyunsaturated fatty acids induced lipid peroxidation, which is a type of oxidative stress, in various types of tissues [7]. Lipid peroxidation is a self-promoting chain reaction targeting polyunsaturated fatty acids in cellular membranes that changes membrane properties and the function of membrane proteins; furthermore, the end-products of lipid peroxidation function as toxic second messengers, inducing apoptosis and necrosis [8, 9]. Some antioxidants can inhibit lipid peroxidation by acting as free radical scavengers, resulting in the formation of non-radical products [10]. It is also well known that propofol itself has antioxidant effects, especially on lipid peroxidation [11, 12].

The purpose of this study was to determine whether it is propofol or its lipid emulsion vehicle that is the cause of the skeletal muscle damage resulting from prolonged infusion of the commercially available propofol formulation. For this, the effects of prolonged intravenous infusion of lipid emulsion and propofol on skeletal muscle were evaluated using a rat model.

## Methods

This manuscript was written in accordance with Enhancing the QUAlity and Transparency Of health Research (EQUATOR) guidelines.

### Animals and instrumentation

The protocol of this study was approved by the Ethics Committee (approval code: 15-046) for Animals of Sapporo Medical University School of Medicine. This animal experiment complied with the Animal Research: Reporting of In Vivo Experiments (ARRIVE) guidelines and adhered to the guidelines for proper conduct of animal experiments issued by both the Japanese Ministry of Education, Culture, Sports, Science and Technology and the Japanese Ministry of Health, Labour and Welfare, and also by the Science Council of Japan. Male *Wistar* rats (150–200 g) were kept at 22–24 °C with a 12-h light–dark cycle. Ad libitum access to food and water was provided. In all the animals, a disposable sterile intravenous catheter was inserted into the left femoral vein under anesthesia with 3% sevoflurane. The free end of the catheter was tunneled through the subcutaneous tissue from the left femoral site to the center of the rats’ back. To prevent the rats from biting the catheter, the free end in the back was then passed into a stainless-steel coil. A syringe pump (Terufusion^®^ infusion pump; Terumo, Tokyo, Japan) was used for drug infusion. During infusion, each rat was isolated in a separate cage and given free access to food and water, as before catheterization. Moreover, all rats, including those who received propofol, were able to breath spontaneously without any airway maintenance.

### Experimental protocol

Forty rats were randomly divided into the NI group (no intervention), Cath group (venous catheterization only), Prop group (receiving 1% propofol (Maruishi, Osaka, Japan) infusion at 10 mg/kg/h), and Lipid group (receiving 10% Lipofundin (B. Braun, Melsungen, Germany) infusion at 100 mg/kg/h) (*n* = 10, each group); in the Prop and Lipid groups, 1% propofol (Maruishi) or Lipofundin, respectively, was infused at 1 mL/kg/h for 72 h. The solvent of 1% propofol (Maruishi) is a 10% lipid emulsion. The infusion rate of 1% propofol was determined based on the results of preliminary experiments, such that the level of sedation in the rats which received a 20 mg/kg/h propofol infusion was too deep to ingest food or water, inducing circulatory collapse and death within 24 h of the start of infusion. The rats need to be able to ingest food and water freely to prevent caloric depletion and dehydration. Thus, 10 mg/kg/h infusion rate of 1% propofol, a dosage not sufficient to induce sedation in rats, was applied in the Prop group. Since Lipofundin consists of 50% long-chain triacylglycerols and 50% medium-chain triacylglycerols, it has a similar composition as the solvent used in the commercially available propofol preparation. After drug infusion for 72 h, the rats were re-anesthetized with 3% sevoflurane for collection of blood and skeletal muscle samples. The blood samples were collected from the left ventricle of the heart immediately after anesthetic induction. The collected blood samples were centrifuged at 12,000*g* for 10 min to extract the plasma, which was subsequently stored at − 80 °C for enzyme-linked immunosorbent assay and enzymatic assay. Gastrocnemius muscle samples of the right hind limb were used for measurement of superoxide production, fixed with 10% formaldehyde solution for immunohistochemistry, and stored at − 80 °C for Western blot analysis.

### Evaluation of level of consciousness

The consciousness level of each group was evaluated in accordance with the methods of the previous studies [13, 14]. To strictly evaluate the status of consciousness/sedation of the rats during drug infusion, two methods were used in the current study. In the first method, four parameters were assessed: the tail pinch reflex, pedal withdrawal reflex in the forelimbs, pedal withdrawal reflex in the hindlimbs, and corneal reflex. When a reflex reaction to stimulation was observed, the score was recorded as zero. In contrast, when no reflex reaction was observed, the score was recorded as one. The total score of the four reflex tests was calculated, and a score of three or more was considered to indicate that the animal was deeply sedated. A score of zero indicated that the rats were awake, and could spontaneously ingest the ad libitum available food and water. In the second method, two parameters were used to assess consciousness: the toe pinch test and righting reflex. In these two tests, the responses were scored on a scale of 1–5 (1: deeply sedated, 5: not sedated). All evaluations of consciousness were performed at 2, 24, 48, and 72 h after the initiation of drug infusion.

### Enzyme-linked immunosorbent assay

The plasma concentration of myoglobin was determined in 20 μL of plasma using the rat myoglobin ELISA kit (Life Diagnostic Inc., West Chester, PA) according to the manufacturer’s instructions. Data were collected spectrophotometrically using a standard 96-well plate reader at a wavelength of 450 nm (Sunrise™ reader, Tecan Group Ltd., Männedorf, Switzerland).

### Enzymatic assay

Plasma concentrations of creatine kinase (CK) were measured in 5 μL of plasma using a Cicali CK Kit (Kanto Chemical Co., Inc., Tokyo, Japan) according to the manufacturer’s instructions. Data were collected spectrophotometrically using a Beckman Coulter AU5400 chemistry analyzer (Beckman Coulter Inc., Brea, CA).

### Western blotting

Total protein was extracted with ice-cold buffer from a stored skeletal muscle sample [15], and the concentrations of protein were determined with a BCA Protein Assay Kit (Thermo Fisher Scientific, Waltham, MA). Data of protein concentrations were collected spectrophotometrically by GENESYS 10S UV–Vis™ (Thermo Fisher Scientific) at a wavelength of 562 nm. Briefly, equal amounts of proteins were separated by 7.5% sodium dodecyl sulfate-polyacrylamide gel electrophoresis (SDS-PAGE) and transferred to a polyvinylidene fluoride membrane (Thermo Fisher Scientific) after hydrophilization using 100% methanol. Membranes were probed to antibodies against malondialdehyde (MDA) (STA-032, 1:1000; Cell Biolabs, Cambridge, UK) and β-actin (#4967, 1:1000; Cell Signaling Technology, Beverly, MA). The 64-kDa band was used for quantitative evaluation of MDA according to the manufacturer’s instructions. Following protein quantification, protein densities were normalized to internal standards on each gel, and expressed relative to β-actin. The internal standard used was an untreated rat gastrocnemius muscle that underwent the same assessment protocol mentioned above. The blots were then analyzed blindly.

### Immunohistochemistry

After extraction of skeletal muscle, the samples were fixed with 10% formaldehyde. Paraffin-embedded skeletal muscle tissue was cut into 3-μm-thick slices. After deparaffinization followed by blocking with 3% hydrogen peroxide and 4% BLOCK ACE (DS Pharma Biomedical, Osaka, Japan), the slices were incubated with primary antibodies against 4-hydroxynonenal (4-HNE) (ab46545, 1:400; Abcam PLC, Cambridge, UK) for 60 min at room temperature. The sections were incubated with Histofine Simple Stain MAX-PO (Nichirei Biosciences, Tokyo, Japan) for rats. Visualization with diaminobenzidine substrate (Bond Polymer Refine Detection; Leica Biosystems, Wetzlar, Germany) was followed by counterstaining with Mayer’s hematoxylin (Bond Polymer Refine Detection; Leica Biosystems). Images were obtained with a BZ9000 fluorescence microscope (Keyence Corp., Osaka, Japan). Five images were randomly captured for each specimen and analyzed using the Image J program with Color Deconvolution plugin for calculating optical density. A previously described procedure was followed to perform optical density calculations [16, 17].

### Measurements of superoxide production

The oxidative fluorescent dye, dihydroethidium (Thermo Fisher Scientific), was used to evaluate levels of superoxide in situ, as described previously [18]. Skeletal muscle cells are permeable to dihydroethidium, and dihydroethidium is oxidized to fluorescent ethidium bromide by superoxide. Dihydroethidium is known to be a specific fluorescent probe for superoxide detection [19]. Unfixed frozen skeletal muscle samples were sliced into 20-μm-thick slices at − 20 °C and placed on glass slides. Dihydroethidium (2 × 10^–6^ M) in phosphate-buffered saline (pH = 7.4) was applied to each sliced sample. The slices were incubated in a light-protected chamber at 37 °C for 20 min. Images were obtained with a ConfoCol3LSM5 10META (ZEISS, Oberkochen, Germany) laser scanning microscope equipped with a DPSS laser. Fluorescence was detected with a 561-nm long-pass filter. The laser settings were identical for acquisition of images from all slices. Five images were randomly captured for each specimen. Captured images were analyzed with the Image J program, and the data were expressed as percentages of the positive areas presented in the field.

### Statistical analysis

Serum concentrations of CK and myoglobin, the expression level of 4-HNE and MDA, and the superoxide production level were statistically analyzed by one-way analysis of variance followed by Tukey’s post hoc test. Normality and equal variance of data among all the groups were confirmed by the Shapiro–Wilk test and *F* test, respectively. Data are presented as means ± standard deviations. All statistical analyses were performed with GraphPad Prism 7.0 software (GraphPad software, La Jolla, CA). A two-tailed *p* value < 0.05 was considered statistically significant.

Sample size was calculated with the G*power 3.1 statistical power analysis program (Heinrich-Heine-University, Düsseldorf, Germany). According to a previous report, a standard deviation of 37.5 U/L and mean difference of 57.9 U/L in CK are indicative of a clinically meaningful difference in CK [20]. As a result, *n* = 10 in each group was considered an adequate sample size, assuming a statistical power of 0.9 at a two-tailed significance level of 0.05.

## Results

### Level of consciousness

Reportedly, a propofol infusion of 44–55 mg**/**kg/h is required for maintenance of anesthesia in rats [21]. To confirm that the rats were not sedated during the 10 mg**/**kg/h propofol infusion, their consciousness level was evaluated. The total scores of all four parameters were zero and the scores of the additional two tests were five at the four evaluation timings in each group. The results indicate that the dose of propofol used in the Prop group did not reach the dose needed for rat sedation. Additionally, all rats were spontaneously breathing and survived during the 72 h of drug infusion.

### Evaluation of skeletal muscle damage

**Fig. 1:**
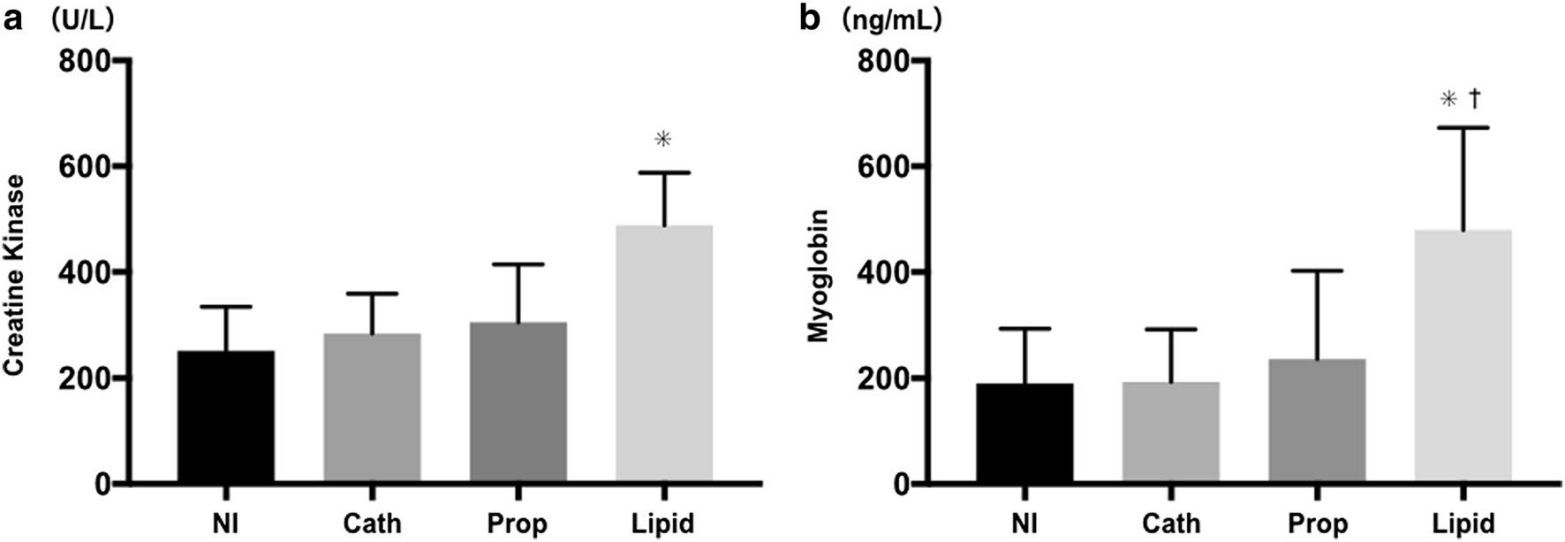
Evaluation of skeletal muscle damage by measuring plasma concentrations of muscle enzymes. **a** Lipid infusion induced elevation of plasma creatine kinase levels. *p < 0.001 compared to each of the other three groups. **b** Lipid infusion induced an elevation of plasma myoglobin levels. *p < 0.001 compared to NI and Cath groups, respectively; ^†^*p* = 0.004 compared to the Prop group. The data are presented as means and standard deviations (*n* = 10)

Plasma concentration of CK in the Lipid group was significantly higher than that in the other three groups (Lipid 489 ± 99 U/L; NI 251 ± 84 [95% CI 126–349] U/L; Cath 284 ± 75 [95% CI 93–316] U/L; Prop 306 ± 109 [95% CI 71–295] U/L, *p* < 0.001 for all). There were no significant differences between the NI, Cath, and Prop groups (Fig. 1a). Similarly, the plasma concentration of myoglobin was also significantly higher in the Lipid group than in the other three groups (Lipid 480 ± 193 ng/mL; NI 109 ± 103 [95% CI 114–466] ng/mL, *p* < 0.001; Cath 193 ± 99 [95% CI 111–463] ng/mL, *p* < 0.001; Prop 236 ± 166 [95% CI 68–420] ng/mL, *p* = 0.004). There were no statistically significant differences between the NI, Cath, and Prop groups (Fig. 1b).

### Superoxide production in lipid peroxidation

**Fig. 2:**
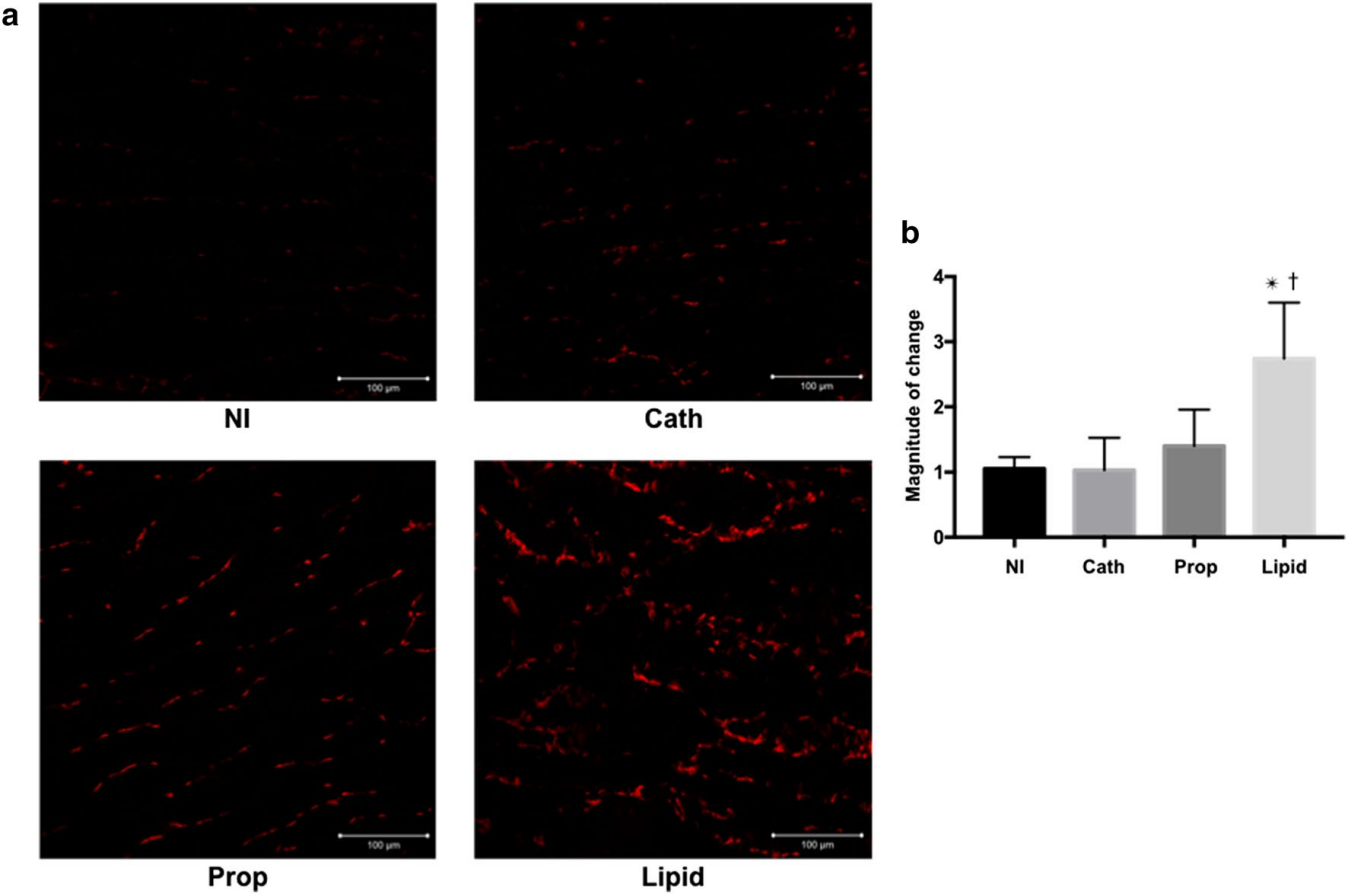
Superoxide production in skeletal muscle detected using dihydroethidium. **a** Representative images of superoxide production in NI, Cath, Prop, and Lipid groups. **b** Quantitative evaluation of superoxide production. *p = 0.001 versus NI and Cath groups, respectively; ^†^*p* = 0.010 versus the Prop group. The data are presented as means and standard deviations (*n* = 5)

Since superoxide is recognized as an intermediate product in lipid peroxidation [19], superoxide production in skeletal muscle was measured using dihydroethidium in this study. Superoxide production was significantly higher in the Lipid group than in the other three groups (Lipid 2.74 ± 0.86; NI 1.05 ± 0.17 [95% CI 0.65–2.73], *p* = 0.001; Cath 1.03 ± 0.49 [95% CI 0.67–2.75], *p* = 0.001; Prop 1.40 ± 0.56 [95% CI 0.30–2.39], *p* = 0.010). There were no statistically significant differences in the other three groups. Quantitative evaluations of superoxide production and representative images for each group are presented in Fig. 2.

### Progression of lipid peroxidation in skeletal muscle

**Fig. 3:**
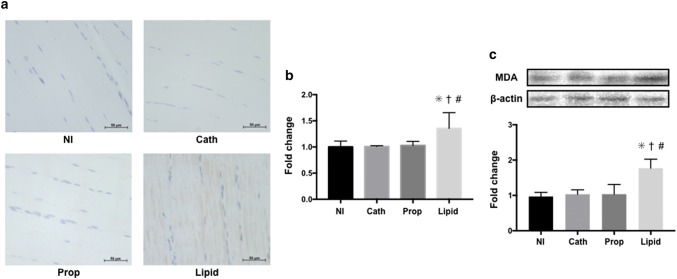
Progression of lipid peroxidation in skeletal muscle. **a** Representative images of immunohistochemistry evaluation of 4-hydroxynonenal in the gastrocnemius muscle. The brown area indicates 4-hydroxynonenal stained by diaminobenzidine. The expression level of 4-hydroxynonenal was higher in the Lipid group than in the other three groups. **b** Quantitative evaluation of the expression level of 4-hydroxynonenal. *p = 0.017 versus the NI group. ^†^*p* = 0.019 versus the Cath group. ^#^*p* = 0.021 versus the Prop group. **c** Representative immunoblot images and the corresponding analysis of the expression of malondialdehyde by Western blotting. *p = 0.010 versus the NI group. ^†^*p* = 0.012 versus the Cath group. ^#^*p* = 0.043 versus the Prop group. The data are presented as means and standard deviations (*n* = 5–6). *MDA* malondialdehyde

To investigate the progression of lipid peroxidation with lipid infusion, expression of 4-HNE and MDA, which are the end products and markers of lipid peroxidation, were evaluated by immunohistochemistry and Western blotting, respectively. The results of immunohistochemistry showed that the expression level of 4-HNE was significantly higher in the Lipid group than in the other three groups (Lipid 1.36 ± 0.30; NI 1.00 ± 0.11 [95% CI 0.06–0.65], *p* = 0.017; Cath 1.01 ± 0.02 [95% CI 0.05–0.64], *p* = 0.019; Prop 1.03 ± 0.08 [95% CI 0.04–0.61], *p* = 0.021, Fig. 3a, b). Furthermore, the expression level of MDA was significantly higher in the Lipid group than in the other three groups (Lipid 1.77 ± 0.25; NI 0.97 ± 0.11 [95% CI 0.30–1.30], *p* = 0.010; Cath 1.03 ± 0.13 [95% CI 0.25–1.22], *p* = 0.012; Prop 1.04 ± 0.27 [95% CI 0.03–1.44], *p* = 0.043, Fig. 3c). These results suggest that lipid infusion causes progressive lipid peroxidation in skeletal muscle.

## Discussion

The main findings of this animal study are that lipid emulsion induces skeletal muscle damage via lipid peroxidation, whereas propofol attenuates lipid-induced skeletal muscle damage.

Lipid peroxidation is a self-promoting chain reaction, in which oxidants attack polyunsaturated fatty acids, resulting in the generation of lipid peroxyl radicals and hydroperoxides, as described previously [9]. Hydroxy radicals and hydroperoxides mainly affect polyunsaturated fatty acids and act as initiators of lipid peroxidation, resulting in the chain reaction. Lipid peroxidation is also induced by intravenous administration of soybean oil-based lipid emulsion, which is rich in polyunsaturated fatty acids [22]. The main end products of lipid peroxidation are MDA and 4-HNE. Both are reliable markers of lipid peroxidation and are considered as toxic second messengers regulating various gene transcriptions and affecting cell proliferation, differentiation, apoptosis, and necrosis [9]. In the present study, lipid infusion induced relatively high expressions of MDA and 4-HNE in skeletal muscle, suggesting that the mechanism of skeletal muscle damage involves lipid peroxidation.

Lipid peroxidation can be terminated by antioxidants. Many studies reported that propofol has antioxidant effects and, in particular, inhibits the lipid peroxidation reaction [11, 23, 24]. Propofol has a similar structure as butylated hydroxytoluene, which is known to be a free radical scavenger, and the anti-lipid peroxidation effect of propofol is larger than that of butylated hydroxytoluene [25]. It is possible that this strong antioxidant effect of propofol contributed to attenuation of the progression of lipid peroxidation and skeletal muscle damage in the Prop group in this study.

The propofol infusion rate in the Prop group in the present study was 10 mg**/**kg/h for 72 h. Bray reported that there was a strong correlation between skeletal muscle damage and a propofol infusion of 4 mg**/**kg/h given for longer than 48 h in a clinical setting [1]. To ensure that the rats survived for more than 48 h, it was necessary for the rats to have free access to water and food and to remain conscious, because management of respiration, circulation, and nutrition is extremely difficult in the sedated state. The infusion rate of propofol for anesthetic maintenance is reportedly 44–55 mg**/**kg/h [26]. Moreover, Schläpfer et al. used propofol for rats and reported that the median infusion rate of propofol was 10.2 mg**/**kg/h [27]. In their study, rats who received propofol remained hemodynamically stable for 24 h. On the basis of these results, the propofol infusion rate of 10 mg**/**kg/h for 72 h was used in the present study to achieve the long duration of propofol infusion.

This study has certain limitations. In the current study, Lipofundin was used in the Lipid group as the vehicle control of 1% propofol (Maruishi) according to the previous reports [28, 29]. Ideally, although a lipid emulsion that is identical to the vehicle of 1% propofol (Maruishi) should have been used in the Lipid group, we were unable to acquire this type of lipid emulsion. Lipofundin is clinically used as a vehicle for propofol in other countries and has the same LCT/MCT ratio (50:50) as the 1% propofol (Maruishi) vehicle. While the fatty acid content of Lipofundin is mentioned as linoleic acid 24–29 mg/mL and α-linolenic acid 2.5–5.5 mg/mL in the manufacturer’s literature, the fatty acid content of the vehicle of 1% propofol (Maruishi) has not been clarified. Linoleic acid, which is one of the n-6 fatty acids, is involved in the inflammatory response and is a regulator of inflammation. On the other hand, α-linolenic acid, which is an n-3 fatty acid, possesses many anti-inflammatory properties. These polyunsaturated fatty acids play important roles in the structure and function of membranes, and serve as substrates for mediators involved in inflammation and the immune response [30]. It is possible that the differences in fatty acid content between Lipofundin and the vehicle of 1% propofol (Maruishi) might have influenced our results. However, previous research that investigated the effects of different intravenous lipid emulsions containing various percentages of linoleic acid and α-linolenic acid on laboratory and clinical outcomes in adult patients reported similar clinical safety profiles and absence of effect on inflammatory markers between various types of lipid emulsions, including a 50:50 mixture of MCTs and soybean oil [29]. Moreover, several studies that compared the effects of soybean oil and a 50:50 mixture of MCTs and soybean oil on immune function reported that there was no difference in immune function [30–32]. We believe that the in vivo effect of the differences in fatty acid contents between 1% propofol (Maruishi) vehicle and Lipofundin is only slight, thus validating our utilization of Lipofundin as a lipid vehicle control. Second, plasma CK or myoglobin levels were not increased in rats receiving propofol, suggesting the possibility that the mechanism of skeletal muscle damage in the present study does not exactly reflect propofol-induced muscle damage. This study used healthy rats as the subjects, while clinically, propofol-induced muscle damage tends to occur in patients who have excessive oxidative stress or mitochondrial dysfunction, such as is seen in sepsis [32]. We believe that the antioxidant effect of propofol is sufficient to suppress lipid peroxidation in a healthy model, although this effect is insufficient under excessive oxidative stress, resulting in skeletal muscle damage. Moreover, we believe that the lipid emulsion should be considered as a possible cause of propofol-induced muscle damage. The third limitation of this study is that hemodynamic parameters could not be evaluated during the drug infusion period, because the drug infusion system physically interfered with the measurement of hemodynamic status. According to a previous report, rats remained hemodynamically stable during a 10 mg**/**kg/h propofol intravenous infusion [27]. Moreover, hemodynamic collapse would be associated with a decrease in level of consciousness of the rats. However, evaluation of the consciousness level of the rats in the current study indicated no deterioration in the consciousness level in any of the rats. Additionally, all rats survived during the 72 h of drug infusion. Thus, it seems that severe hypotension or bradycardia did not occur in this animal model.

In conclusion, the present findings suggest that a 72-h infusion of lipid emulsion induces skeletal muscle damage via lipid peroxidation, while propofol itself attenuates muscle damage by its antioxidant effect in healthy adolescent rats. Further studies should be conducted to clarify whether the lipid emulsion contributes to skeletal muscle damage in clinical settings.

## References

[1] Bray RJ (1998). Propofol infusion syndrome in children. Paediatr Anaesth.

[2] Hatch DJ (1999). Propofol-infusion syndrome in children. Lancet.

[3] Vanlander AV, Okun JG, de Jaeger A, Smet J, De Latter E, De Paepe B, Dacremont G, Wuyts B, Vanheel B, De Paepe P, Jorens PG, Van Regenmortel N, Van Coster R (2015). Possible pathogenic mechanism of propofol infusion syndrome involves coenzyme q. Anesthesiology.

[4] Branca D, Roberti MS, Vincenti E, Scutari G (1991). Uncoupling effect of the general anesthetic 2,6-diisopropylphenol in isolated rat liver mitochondria. Arch Biochem Biophys.

[5] Rigoulet M, Devin A, Avévet N, Vandais B, Guérin B (1996). Mechanisms of inhibition and uncoupling of respiration in isolated rat liver mitochondria by the general anesthetic 2,6-diisopropylphenol. Eur J Biochem.

[6] Carpentier YA, Dupont IE (2000). Advances in intravenous lipid emulsions. World J Surg.

[7] Pitkänen O, Hallman M, Andersson S (1991). Generation of free radicals in lipid emulsion used in parenteral nutrition. Pediatr Res.

[8] Yin H, Xu L, Porter NA (2011). Free radical lipid peroxidation: mechanisms and analysis. Chem Rev.

[9] Zarkovic N (2003). 4-hydroxynonenal as a bioactive marker of pathophysiological processes. Mol Aspects Med.

[10] Halliwell B, Gutteridge JM (1984). Oxygen toxicity, oxygen radicals, transition metals and disease. Biochem J.

[11] Runzer TD, Ansley DM, Godin DV, Chambers GK (2002). Tissue antioxidant capacity during anesthesia: propofol enhances in vivo red cell and tissue antioxidant capacity in a rat model. Anesth Analg.

[12] Murphy PG, Myers DS, Davies MJ, Webster NR, Jones JG (1992). The antioxidant potential of propofol (2,6-diisopropylphenol). Br J Anaesth.

[13] Kawai S, Takagi Y, Kaneko S, Kurosawa T (2011). Effect of three types of mixed anesthetic agents alternate to ketamine in mice. Exp Anim.

[14] Perez-Zoghbi JF, Zhu W, Grafe MR, Brambrink AM (2017). Dexmedetomidine-mediated neuroprotection against sevoflurane-induced neurotoxicity extends to several brain regions in neonatal rats. Br J Anaesth.

[15] Ishikawa A, Ogawa K, Tokinaga Y, Uematsu N, Mizumoto K, Hatano Y (2007). The mechanism behind the inhibitory effect of isoflurane on angiotensin II-induced vascular contraction is different from that of sevoflurane. Anesth Analg.

[16] Ruifrok AC, Johnston DA (2001). Quantification of histochemical staining by color deconvolution. Anal Quant Cytol Histol.

[17] Varghese F, Bukhari AB, Malhotra R, De A (2014). IHC Profiler: an open source plugin for the quantitative evaluation and automated scoring of immunohistochemistry images of human tissue samples. PLoS ONE.

[18] Nakahata K, Kinoshita H, Azma T, Matsuda N, Hama-Tomioka K, Haba M, Hatano Y (2008). Propofol restores brain microvascular function impaired by high glucose via the decrease in oxidative stress. Anesthesiology.

[19] Wardman P (2007). Fluorescent and luminescent probes for measurement of oxidative and nitrosative species in cells and tissues: progress, pitfalls, and prospects. Free Radic Biol Med.

[20] Minetto MA, Lanfranco F, Botter A, Motta G, Mengozzi G, Giordano R, Picu A, Ghigo E, Arvat E (2011). Do muscle fiber conduction slowing and decreased levels of circulating muscle proteins represent sensitive markers of steroid myopathy? A pilot study in Cushing’s disease. Eur J Endocrinol.

[21] Glen JB, Hunter SD (1984). Pharmacology of an emulsion formulation of ICI 35 868. Br J Anaesth.

[22] Kellogg KW, Fridovich I (1975). Superoxide, hydrogen peroxide, and singlet oxygen in lipid peroxidation by a xanthine oxidase system. J Biol Chem.

[23] Pitkänen OM, Luukkainen P, Andersson S (2004). Attenuated lipid peroxidation in preterm infants during subsequent doses of intravenous lipids. Biol Neonate.

[24] Esterbauer H, Eckl P, Ortner A (1990). Possible mutagens derived from lipids and lipid precursors. Mutat Res.

[25] Esterbauer H, Schaur RJ, Zollner H (1991). Chemistry and biochemistry of 4-hydroxynonenal, malonaldehyde and related aldehydes. Free Radic Biol Med.

[26] Green TR, Bennett SR, Nelson VM (1994). Specificity and properties of propofol as an antioxidant free radical scavenger. Toxicol Appl Pharmacol.

[27] Schläpfer M, Piegeler T, Dull RO, Schwartz DE, Bonini MG, Z’Graggen BR, Beck-Schimmer B, Minshall RD (2015). Propofol increases morbidity and mortality in a rat model of sepsis. Crit Care.

[28] Knibbe CA, Aarts LP, Kuks PF, Voortman HJ, Lie-A-Huen L, Bras LJ, Danhof M (2000). Pharmacokinetics and pharmacodynamics of propofol 6% SAZN versus propofol 1% SAZN and Diprivan-10 for short-term sedation following coronary artery bypass surgery. Eur J Clin Pharmacol.

[29] Jones CJ, Calder PC (2018). Influence of different intravenous lipid emulsions on fatty acid status and laboratory and clinical outcomes in adult patients receiving home parenteral nutrition: a systematic review. Clin Nutr.

[30] Miles EA, Calder PC (2015). Fatty acids, lipid emulsions and the immune and inflammatory systems. World Rev Nutr Diet.

[31] Sedman PC, Somers SS, Ramsden CW, Brennan TG, Guillou PJ (1991). Effects of different lipid emulsions on lymphocyte function during total parenteral nutrition. Br J Surg.

[32] Gogos CA, Kalfarentzos FE, Zoumbos NC (1990). Effects of different types of total parenteral nutrition on T-lymphocyte subpopulations and NK cells. Am J Clin Nutr.

